# Prognostic value of serum proadrenomedullin in catheter-related bloodstream infection in the intensive care unit: A prospective observational study

**DOI:** 10.1097/MD.0000000000012821

**Published:** 2018-10-19

**Authors:** Juping Ni, Yingjie Sun, Hongping Qu, Aqian Wang, Yunshan Cao, Xiang Li

**Affiliations:** a Department of Intensive Care, Minhang Hospital, Fudan University; b Department of Critical Care Medicine, Ruijin Hospital, Shanghai Jiaotong University School of Medicine, Shanghai; c Department of Cardiology, Gansu Provincial Hospital, Lanzhou, Gansu, People's Republic of China.

**Keywords:** catheter-related bloodstream infection, proadrenomedullin, procalcitonin, prognostic value

## Abstract

Patients with catheter-related bloodstream infection (CRBSI) have a poor prognosis. Proadrenomedullin (pro-ADM) has emerged as a valuable marker of sepsis. The potential role of pro-ADM in predicting the prognosis of CRBSI was evaluated. We enrolled 25 CRBSI patients and pro-ADM level was measured within 24 hours after each admission. Survival was assessed after 28 days. Among 25 patients with CRBSI, 14 patients survived. Pro-ADM in survivors was significantly lower than that in non-survivors (3.71 ± 1.30 vs 5.58 ± 1.18 nmol/L). The area under the curve (AUC) for pro-ADM was 0.87 (95% CI 0.68–0.97) with a cut-off value of 4.67 nmol/L, providing sensitivity of 85.7% and specificity of 81.8%. The AUCs for PCT, WBC, and CRP were 0.76 (95% CI 0.55–0.90), 0.72 (95% CI 0.50–0.88), and 0.69 (95% CI 0.48–0.86), respectively. Kaplan–Meier survival curves showed pro-ADM ≥ 4.67 nmol/L was associated with higher mortality (log-rank p = 0.001). Moreover, the pro-ADM level was significantly higher in patients with septic shock than those without shock (5.44 ± 1.17 vs 3.54 ± 1.18nmol/L). The mortality of patients with septic shock was higher than that of patients without shock (69.2% vs 16.7%, *P* = .008). In conclusion, pro-ADM could be used as a prognostic marker of CRBSI in critically ill patients.

## 1 Introduction

Central venous catheters (CVCs) are widely used in intensive care units (ICU).^[1]^ However, CVCs are associated with some complications in clinical practice.^[2]^ Catheter-related bloodstream infection (CRBSI) is a common serious complication, which can cause significantly longer ICU and hospital stays with higher total mortality rate and hospital cost.^[3,4]^

Some biomarkers have been proposed to assess the severity and prognosis of systemic inflammation, infection, and sepsis, such as white blood cell (WBC) count, C-reactive protein (CRP), and procalcitonin (PCT), in critically ill patients.^[5,6]^ Therefore, these biomarkers have been assessed for the early prediction of patients with CRBSI; however, they have shown only limited sensitivity and specificity for the prognosis of CRBSI.^[7,8]^

Proadrenomedullin (pro-ADM) is a stable fragment of adrenomedullin (ADM) with immune modulation, vasodilatation, and antimicrobial activity^[9]^ during severe infections. Published data have shown that pro-ADM can be used for risk assessment in patients with sepsis and bloodstream infection.^[10]^ However, there is limited data about the prognostic value of pro-ADM in adult patients with CRBSI, which the present study was designed to evaluate.

## 2 Materials and methods

### 2.1 Patients

After approval from the Research Ethics Committee of Minhang Hospital, Fudan University and written informed consent from each patient were obtained, 25 CRBSI patients (mean age 70.76 years [SD 11.9] and 64.0% male) from January 2012 to August 2014 were enrolled in this prospective, observational, nonrandomized study. All methods were performed in accordance with the relevant guidelines and regulations, and the guidelines^[21]^ were also followed to treat all patients with either severe sepsis or septic shock in this study. Pro-ADM testing was performed free of charge for all patients. Also, it is cost effective.

All patients were empirically treated with antibiotics according to local antimicrobial susceptibility data. Inclusion criteria were

**Figure 1:**
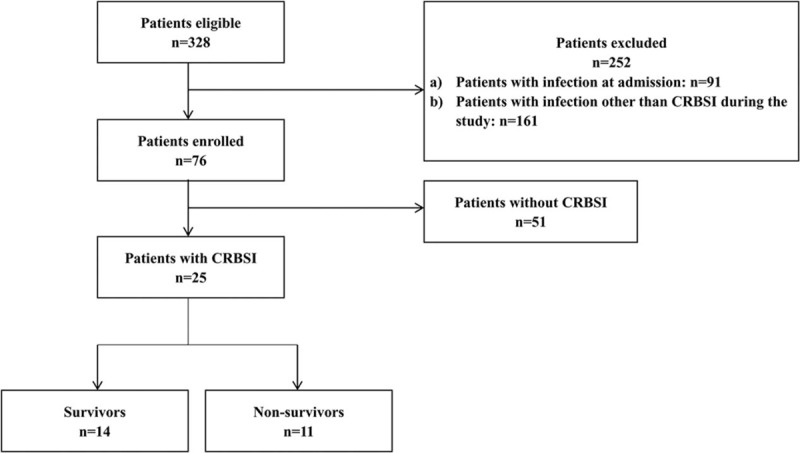
Flow diagram for patients enrolment or exclusion in the study. CRBSI: Catheter – related bloodstream infection.

1) age ≥ 18 years;
2) CVC in place for more than 48 hours; and
3) clinical manifestations of infection (e.g., fever, chills, and/or hypotension).^[1]^ Exclusion criteria were infection at admission and infection other than CRBSI during the study. Three hundred twenty-eight patients admitted to the ICU were included in the study. CRBSI was defined according to published guidelines.^[1]^ Patients with CRBSI were divided into 2 groups according to 28-day mortality: survivors and non-survivors (Fig. 1)

### 2.2 Data collection

Clinical data were collected within 24 hours after enrollment by an experienced physician. For each patient, the following information was recorded: age, sex, underlying diseases, comorbidity, mean arterial pressure, need for ICU admission, laboratory findings including WBC count, CRP, PCT, and serum creatinine. Acute physiology and chronic health evaluation (APACHE) II score of illness severity was calculated upon enrollment. Clinical outcomes and in-hospital mortality were assessed.

### 2.3 Pro-ADM procedure

Blood samples were collected in serum separator tubes. All samples clotted for 2 hours at room temperature (22°C) before centrifugation for 15 minutes at 1000 × g. Then the serum was removed and stored at −80°C until it was assayed. In order to avoid measurement bias, the pro-ADM level was determined by quantitative sandwich enzyme immunoassay technique as extensively described,^[11]^ according to the manufacturer's recommendations (all kits from CUSABIO, China). The median concentration of pro-ADM in a cohort of healthy individuals was 0.39 nmol/L (97.5th percentile: 0.55 nmol/L).^[12]^

### 2.4 Endpoints

The primary endpoint was 28-day mortality. The second endpoint was presence of septic shock after admission.

### 2.5 Statistical analyses

Prospective power analysis based on pilot data showed that 9 subjects per group would have an 80% power to detect a mean difference of 1.8 nmol/L in pro-ADM level, the primary outcome variable of this study, assuming that the common standard deviation (SD) was 1.3 using a 2-group *t* test with a 2-sided significance level of 0.05. The data were analyzed using SPSS for Windows (version 14; IBM Corporation, Armonk, NY). The Kolmogorov–Smirnov test was used to test the normality of the data recorded. Normally distributed data are presented as mean ± SD, other data are presented as median (range). Student *t* test or Mann–Whitney *U* test was used to test the differences between the 2 study groups. Categorical variables were presented as frequencies and percentages (%) and analyzed by the chi-square test. A receiver operating characteristics curve (ROC) was constructed to evaluate the prognostic significance of the tested parameters. Sensitivity, specificity, and positive and negative predictive values were calculated for each cut-off value of the biomarkers for predicting the primary endpoint. Kaplan–Meier survival curves were generated to illustrate survival probability and clinical outcome for different levels of pro-ADM. A *P* value of 0.05 was considered statistically significant.

## 3 Results

### 3.1 Characteristics of patients on admission

A total of 25 CRBSI patients were enrolled in this study. The mean age was 70.76 years (SD 11.9), and 64.0% were male. The demographic and clinical characteristics of patients on admission and final outcome are presented in Table 1.

**Table 1:**
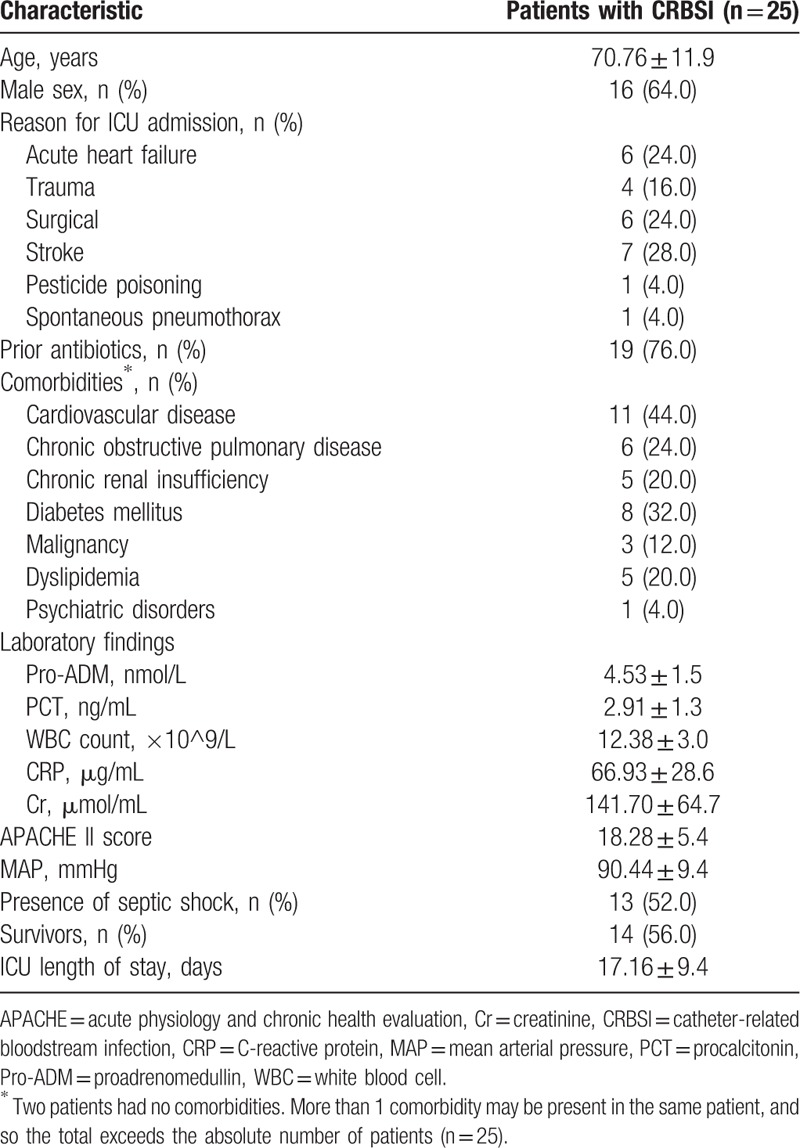
Demographic and clinical characteristics of 25 patients with CRBSI.

### 3.2 Serum pro-ADM levels and other biomarkers in survivor and non-survivor group

Serum levels of pro-ADM, PCT, WBC, and CRP in the 2 groups are presented in Table 2. In the survival group the levels of pro-ADM, PCT, and WBC were significantly lower than those in the non-survival group (all *P* < .05), but CRP concentration did not significantly differ between the 2 groups (*P* > .05).

**Table 2:**
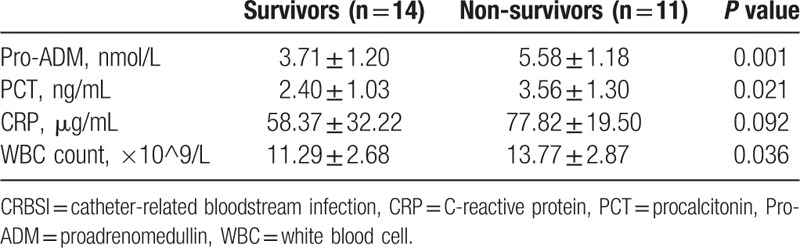
Serum concentration of studied biomarkers in 25 CRBSI patients.

### 3.3 Prognostic significance—ROC analysis

The area under curve (AUC) was investigated for the prognostic accuracy of all studied biomarkers of patients with CRBSI.The area under curve (AUC) for pro-ADM, 0.87 (95% CI: 0.68–0.97), was significantly higher than 0.76 for PCT (95% CI: 0.55–0.90*; P* = .037), 0.72 for WBC (95% CI: 0.50–0.88; *P* = .027), and 0.69 for CRP (95%CI: 0.48–0.86; *P* = 0.014). Furthermore, the optimal cut-off values for pro-ADM (4.67nmol/L) was defined for the prognosis of CRBSI, with sensitivity of 85.7% and specificity of 81.8%. The cut-off values for PCT, CRP serum levels, and WBC were 3.23ng/mL, 54.32 μg/mL, and 12.43∗10^9/L respectively, while the sensitivities and specificities for PCT (92.9%, 63.6%), CRP (71.4%, 72.7%), and WBC (57.1%, 60.9%) were determined, respectively.

### 3.4 Serum pro-ADM in predicting outcome and septic shock

**Figure 2:**
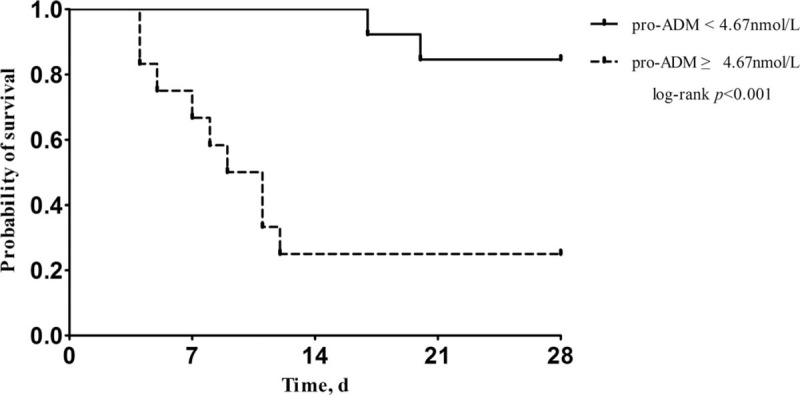
Comparison of survival between patients with different pro-ADM levels.

The primary outcome occurred in 11 of 25 patients with CRBSI (44.0%) within 28 days after enrollment in the ICU. Serum pro-ADM ≥4.67 nmol/L was associated with higher mortality (log-rank *P* < .001) (Fig. 2). Thirteen patients (52.0%) with CRBSI who progressed to a state of septic shock demonstrated significantly increased pro-ADM levels compared with patients without septic shock (5.44 ± 1.17 nmol/L vs 3.54 ± 1.18 nmol/L, *P* = .001). The mortality of patients with septic shock was also significantly higher than that of patients without septic shock (69.2% vs 16.7%, *P* = .008).

## 4 Discussion

This prospective observational study was designed to investigate the clinical utility of pro-ADM in comparison to PCT and CRP in critical patients with CRBSI. We found that the serum pro-ADM level was lower in the survival group than that in the non-survival group, and the AUC for pro-ADM was significantly higher than those for PCT, WBC, and CRP. We also demonstrated that serum pro-ADM ≥ 4.67 nmol/L was associated with higher mortality, and that pro-ADM was significantly increased in patients with CRBSI progressing to septic shock.

In the survival group, the level of pro-ADM was significantly lower than that in the non-survival group. There are no comparable data in the literature, but we believe the increase of pro-ADM in the non-survival group is due to severe infection-related changes in many tissues. CRBSI can cause SIRS and even lead to septic shock.^[13]^ Pro-ADM, as a calcitonin gene-related peptide, is generally upregulated during SIRS or sepsis because bacterial endotoxins and pro-inflammatory cytokines upregulate the ADM gene.^[14]^ Therefore, the increased level of serum pro-ADM in our CRBSI subjects may be explained in part by ADM gene upregulation associated with CRBSI.

As expected, pro-ADM level was also significantly increased in patients with CRBSI who developed septic. In addition, the mortality of patients with septic shock was significantly higher than that of patients without septic shock. These findings are consistent with previous reports that plasma ADM levels are markedly increased in patients with septic shock.^[15–18]^ Furthermore, Hirata and colleagues^[19]^ have demonstrated a correlation between renal function and the pro-ADM level in the infections, suggesting that the increased pro-ADM in sepsis may be due in part to decreased clearance with declining kidney function. Moreover, it was reported that lung injury owing to sepsis might impair the removal of circulating ADM,^[20]^ which also may contribute to an increase of pro-ADM level. Septic shock is an infection-induced syndrome that frequently results in multiple organ dysfunction syndromes.^[21]^ As a result, the decreased renal and pulmonary function in the CRBSI subjects with septic shock in our study (unpublished data) may contribute to an elevation in the pro-ADM level.

This study also highlights some prognostic implications of pro-ADM measurement in critically ill patients. Plasma pro-ADM was previously found to be significantly higher in non-survivors with septic shock than in survivors and to demonstrate better mortality prediction in septic shock patients.^[16–18]^ These findings are consistent with the present study. However, the pro-ADM levels in previous studies^[16,17,22,23]^ were analyzed during the course of sepsis due to respiratory,^[16,22]^ urinary tract,^[16,23]^ or abdominal infections,^[16,17]^ but not CRBSI as evaluated in the present study. It was identified a cut-off point of 4.67 nmol/L for pro-ADM as predictive of a worse outcome after suffering CRBSI as reflected by 28-day mortality. Previously biomarkers of inflammation (WBC, CRP) were used to evaluate the diagnosing CRBSI during the clinical practice, but their specificity is relatively limited.^[8,24]^ Furthermore, patients with higher serum pro-ADM levels could be predicted to have a worse survival rate, which suggests that pro-ADM may be a better inflammatory biomarker for predicting the outcome of critically ill patients with CRBSI. It is shown more sensitive and specificity of pro-ADM in the present study, we therefore believe that this technique may be considered as a parameter to remove central catheters and guiding antibiotic therapy in critically ill patients, and further research is warranted.

Our results must be interpreted after considering the following facts in this sequential analysis study. Only 25 critically ill patients with CRBSI were included in our study, the absolute number is relatively smaller although its sample size had been calculated on the pilot data. Additionally, it was an observational study, and not an interventional study, therefore, the sample population should have no significant impact on the findings. Second, it was unable to create the desired groups in the withdrawal of all clinical confounders of the critically ill subjects in the ICU for practical reasons. Moreover, patients with any other clear source of infections were also excluded on admission in this study. Thus, we believe our findings are unlikely to have been influenced by the potential confounders, and further research in the larger population is warranted.

In conclusion, serum pro-ADM was found to be significantly higher in non-surviving patients with CRBSI than in survivors. Pro-ADM may be a promising inflammatory biomarker for predicting the outcome of critically ill patients with CRBSI.

## 5 Limitations

Our study has several limitations. It was a single-center observational study, and the sample population was relatively small and heterogeneous regarding the underlying disease conditions, thus center-specific differences may be controlled in a multi-center study. Its generalizability is also limited by the fact that only 25 critically ill patients were included on admission in our study. A control group was not designed, and 2 groups including proven CRBSI and unproven CRBSI from the patients with CVC were categorized to create the desired groups, and patients who had any other infection were excluded during this study, our results are preliminary, and a control population without central lines may improve a better understanding of other blood stream infections but it may increase the more strength of the present findings about biomarkers and financial constraints. However, more patients and daily follow-up measurements are warranted in future studies. Moreover, beyond this first analysis in our study, we further also investigated the potential of pro-ADM to independently predict for antibiotic therapy.

## Author contributions

**Conceptualization:** Xiang Li.

**Data curation:** Juping Ni, Hongping Qu.

**Formal analysis:** Juping Ni.

**Funding acquisition:** Xiang Li.

**Methodology:** Yingjie Sun, Aqian Wang.

**Project administration:** Xiang Li.

**Software:** Yingjie Sun.

**Supervision:** Xiang Li.

**Writing – original draft:** Juping Ni, Xiang Li.

**Writing – review & editing:** Yunshan Cao, Xiang Li.
